# Formate simultaneously reduces oxidase activity and enhances respiration in *Campylobacter jejuni*

**DOI:** 10.1038/srep40117

**Published:** 2017-01-16

**Authors:** Issmat I. Kassem, Rosario A. Candelero-Rueda, Kawthar A. Esseili, Gireesh Rajashekara

**Affiliations:** 1 Food Animal Health Research Program, Ohio Agricultural Research and Development Center, Department of Veterinary Preventive Medicine, The Ohio State University, Wooster, OH 44691, USA

## Abstract

The foodborne microaerophilic pathogen, *Campylobacter jejuni,* possesses a periplasmic formate dehydrogenase and two terminal oxidases, which serve to metabolize formate and facilitate the use of oxygen as a terminal electron acceptor, respectively. Formate, a primary energy source for *C. jejuni*, inhibits oxidase activity in other bacteria. Here, we hypothesized that formate might affect both energy metabolism and microaerobic survival in *C. jejuni*. Subsequently, we showed that *C. jejuni* 81–176 (wildtype) exhibited enhanced chemoattraction to and respiration of formate in comparison to other organic acids. Formate also significantly increased *C. jejuni*’s growth, motility, and biofilm formation under microaerobic (5% O_2_) conditions. However, formate reduced oxidase activity under microaerobic conditions as well as aerotolerance and biofilm formation under ambient oxygen conditions. The expression of genes encoding the ribonucleotide reductase (RNR) and proteins that facilitate the use of alternative electron acceptors generally increased in the presence of formate. Taken together, formate might play a role in optimizing *C. jejuni*’s adaptation to the oxygen-limited gastrointestinal tract of the host. By affecting oxidase activity, formate possibly facilitates shuttling electrons to alternative acceptors, while likely conserving limited oxygen concentrations for other essential functions such as DNA synthesis via RNR which is required for *C. jejuni*’s growth.

*Campylobacter jejuni*, a foodborne pathogen, is associated with gastroenteritis and rare but serious post-infection complications in humans^1^. Although *C. jejuni* infections are commonly self-limiting, the ability of this pathogen to persist in food animals and to contaminate cognate food products poses a serious challenge for food safety and global health^2^. Furthermore, in developed nations, infections associated with *C. jejuni* exert a considerable economic burden. For example, in the USA, it was estimated that *Campylobacter*-associated infections cost $1.7 billion and result in the loss of 13,300 quality-adjusted life years (QALYs)^3^. Despite significant progress in understanding the pathobiology of *C. jejuni*, many factors that promote this pathogen’s ability to colonize and/ or infect the gastrointestinal (GI) tract of its hosts remain not completely understood.

The mammalian GI tract is complex and encloses niches with diverse biological and physiochemical properties, including differences in pH (ranges between 4.0 and 8.5), hormones, enzymes, metabolites, and resident microbial communities. These factors can significantly influence the behavior and fate of a potential pathogen in the GI tract. For example, microbial metabolites such as short-chain fatty acids (SCFA), succinate, hydrogen, and secondary bile acids have been shown to affect the growth and virulence of gut pathogens^4^. SCFAs are produced as byproducts of the metabolism of resident intestinal bacteria and the breaking down of foods^5^,^6^. It has been reported that SCFAs are involved in the regulation of virulence genes in certain pathogens^5^,^7^. For example, formate, a SCFA, can act as a signal to induce *Salmonella* invasion of the small intestine in mice^5^. Specifically, formate impacted the major regulators of *Salmonella* pathogenicity island 1 (SPI1) which encodes components of the type III secretion system and other proteins that play a role in the entry to eukaryotic cells^5^. Studies in mice also showed that the presence of formate was a factor in determining the distal ileum as the preferred site for *Salmonella* invasion^5^. Furthermore, formate repressed proteins in the RpoS regulon of *E. coli*^8^, while relatively low concentrations of formate protected both *E. coli* and *Salmonella* from killing by a cationic antimicrobial peptide, which was derived from the human bactericidal/permeability-increasing protein (BPI)^9^. Similarly, it has been shown that formate plays an important role in *C. jejuni*’s pathobiology. For example, several studies reported that disparate *C. jejuni* mutant strains that were deficient in formate metabolism exhibited: (1)- reduced ability to infect human intestinal cells as well as primary chicken intestinal cells *in vitro*^10^,^11^, (2)- deficiency in the colonization of 1-day old chickens^12^,^13^, and (3)- impairments in motility, resistance to oxidative stress, and biofilm formation and defects in cell shape^11^. This is not surprising, because *C. jejuni* exhibits a strong chemoattraction towards formate^14^,^15^, which is also a major energy source for the bacterium^16^.

*C. jejuni* possesses a periplasmic formate dehydrogenase (FdhABCD), which facilitates the breakdown of formate into carbon dioxide and water^12^,^13^,^17^. Electrons generated from the oxidation of formate are passed down a branching electron transport chain, which includes a quinone pool, a cyctochrome *bc*_*1*_ complex, periplasmic *c-*type cytochromes, two terminal oxidases (CioAB: a cyanide-insensitive oxidase and CcoNOQP: a *cb*-type oxidase) and other respiratory proteins that facilitate the use of alternative terminal electron acceptors^12^,^18^. Terminal oxidases enable the use of oxygen as a terminal electron acceptor, which allows *C. jejuni* to persist under low oxygen stress and maintain its microaerobic lifestyle^12^,^19^. Therefore, electrons produced from formate metabolism can contribute to both energy production and survival under microaerobic conditions. Interestingly, oxidase activity can be inhibited by formate in other bacteria^20^,^21^. At first, this contrasted with the aforementioned advantages associated with formate metabolism in *C. jejuni*, especially because an oxidase mutant was unable to colonize the GI tract of chickens^22^. To resolve this paradox, we hypothesized that formate acts to balance energy metabolism and microaerobic survival, likely to allow the use of limited oxygen in the gut for other vital functions. Notably, it has been shown that oxygen is crucial for DNA synthesis^23^ in *C. jejuni* and is considered an important respiratory acceptor in the chicken gut^22^. The latter observation was based on the inability of an oxidase mutant (*ccoN*::Cm strain) to colonize chickens^22^. Therefore, here we investigated the impact of formate on oxidase activity and monitored cognate respiratory activity of *C. jejuni* under microaerobic (5% O_2_) and more restrictive oxygen conditions (<1% O_2_).

## Materials and Methods

### *C. jejuni* strains and culture conditions

*C. jejuni* 81–176 (wildtype)^24^ was cultured on Mueller-Hinton (MH) agar at 42 °C under microaerobic conditions (85% N_2_, 10% CO_2_, 5% O_2_) or under <1% O_2_ (hereafter referred to as anaerobic conditions) which was generated using the BD GasPak Sachets system (BD Diagnostics, NJ, USA)^11^. The *Campylobacter* selective supplement (SR155E, Oxoid, KS, USA), sodium formate, sodium succinate, sodium fumarate, sodium cyanide, sodium azide and sodium nitrate (Sigma, MO, USA) were added to the MH medium when necessary.

### BIOLOG and chemotaxis assays

BIOLOG-AN plates (BIOLOG Inc., CA, USA) contain important metabolic substrates, and the metabolism of these substrates by bacteria results in the production of purple formazan which can be quantified spectrophotometrically at 550 nm. As described in Brandl *et al*.^25^ and Kassem & Rajashekara^17^, *C. jejuni* was grown on laked horse-blood agar for 24 h. The cultures were then suspended in 10 mM phosphate buffer (pH 7.0), centrifuged, washed twice, and resuspended to achieve an OD_600_ of 0.7. The suspensions were transferred to the BIOLOG-AN plates (100 μL/well) which were then incubated microaerobically at 42 °C for 48 h and the OD_550_ was measured. Wells that did not contain any substrates were used as negative controls. The experiment was repeated three times.

Chemotaxis assay was performed as described in Zhulin *et al*.^26^. This method relies on quantifying the change in optical density of bacterial suspension in response to a chemical gradient of the tested substrates which included formate, succinate, fumarate and other compounds. Different chemicals (potential chemoattractants at 100 mM final concentration, Sigma) were suspended in equal volume of molten 2% agarose gel. Suspensions (300 μL) were added carefully to the bottom of plastic cuvettes and were left for 15 minutes to solidify and form agarose plugs. The volume of the plug was selected to ensure that the lower limit of the light beam from a spectrophotometer would be just above the upper edge of the plug. *C. jejuni* cultures were grown on MH agar plates under microaerobic conditions at 42 °C. The cultures were harvested, washed twice and suspended in Adler chemotaxis buffer [10 mM potassium phosphate and 0.1 mM EDTA (pH 7.0)] to achieve an OD_600_ of 0.02. One mL of the bacterial suspension was added on top of the agarose plugs containing the different chemicals. Changes in the OD_600_ of the bacterial suspension in response to the chemical gradient were quantified using a spectrophotometer. An agarose plug containing 1X PBS was used as a negative control and to assess chemotaxis-independent bacterial sedimentation. The samples were tested in triplicate and the experiment was repeated three times.

### *C. jejuni*’s growth, motility and biofilm formation

To evaluate the impact of formate on *C. jejuni*’s growth, a formate solution (5 M) was freshly prepared by dissolving sodium formate in autoclaved and deionized water, which was followed by filtration using 0.2 μm sterile syringe filters (EMD Millipore, USA). *C. jejuni* was then inoculated into MH broth and adjusted to an OD_600_ of 0.1, and formate (0, 5, 10 mM final concentration) was immediately added to the cultures. Aliquots (180 μL) from each culture were transferred to a sterile 96-well plate, which was incubated with shaking (180 rpm) under microaerobic conditions at 42 °C. Growth was monitored at different time-points by measuring OD_600_^13^ using a microplate reader (SpectraMax Plus, Molecular Devices, USA). Growth was further quantified at the time-point (36 h) that reflected the highest significant difference in the OD_600_ between the cultures. For this purpose, the cultures were serially diluted (10-fold), which was followed by spreading aliquots (100 μL) on MH agar plates, and counting colony forming units (CFU).

The impact of formate on motility and biofilm formation was determined as described previously^11^,^27^. To evaluate motility, *C. jejuni* cultures were adjusted to OD_600_ of 0.02. Two μl of each culture were then stabbed into semisolid MH plates containing 0.4% agar and different concentrations of formate (0, 5, 10, 25, and 50 mM). The plates were incubated at 42 °C under microaerobic conditions. Diameters of the zones of motility were measured after 48 h of incubation.

Biofilm formation was assessed by using crystal violet staining^11^,^27^. *C. jejuni* was suspended in MH broth containing formate (0, 5, 10, 25 and 50 mM) to achieve an OD_600_ of 0.05. One ml of culture was transferred to sterile borosilicate glass tubes, which were incubated for 72 h under microaerobic, anaerobic, and aerobic (ambient oxygen) conditions, respectively. The tubes were then gently washed with distilled water and stained with 0.1% crystal violet solution for 15 min. After further washing to remove excess stain, the tubes were left to dry at room temperature. The biofilms were then dissolved in 80% DMSO and quantified spectrophotometrically (OD_570_). The effect of different concentrations of sodium fumarate and sodium succinate on motility and biofilm formation was also assessed as described above. Fumarate and succinate were included as controls to contrast with the specific impact of formate on these phenotypes. All experiments were repeated at least three times and samples were tested in triplicate.

### Respiratory activity

*C. jejuni*’s respiratory activity in the presence of formate was measured using 5-cyano-2,3-ditolyl tetrazolium chloride (CTC) staining as described elsewhere^28^,^29^. CTC acts as an artificial final electron acceptor, which is reduced into a chromogenic product (formazan)^29^. Briefly, *C. jejuni* cultures were adjusted to OD_600_ of 0.2 in MH broth. Different concentrations of formate were added to the cultures as described above. The cultures were then incubated shaking (200 rpm) under microaerobic conditions at 42 °C. After 30 min, 900 μL was aliquoted from each culture and 100 μL of CTC (~5 mM final concentration) was added. The samples were then mixed thoroughly and incubated in the dark at room temperature for 45 min. Colorimetric CTC reduction (respiration) was determined using a spectrophotometer (OD_550_). Respiratory activity was also conducted under anaerobic conditions in the presence and absence of formate and/or nitrate (1 mM final concentration). The latter was added to assess respiration in the presence of an alternative electron acceptor. In order to reduce any compromise of the anaerobic conditions, these experiments were conducted in 96-well plates using 90 μL of culture and 10 μL of CTC for each reaction. In all experiments, sterile MH broth with CTC served as a negative control. Different concentrations of fumarate and succinate were included as additional controls to contrast with the specific impact of formate on respiration. The samples were tested in triplicate and the experiment was repeated three times.

### Oxidase activity

Oxidase activity was measured using N,N,N′,N′-tetramethyl-p-phenylenediamine (TMPD)^30^. *C. jejuni* cultures were adjusted to OD_600_ of 0.2 in MH broth which was supplemented with formate as described above. After incubation for 30 min, 900 μL of each culture were used to assay for oxidase activity by adding 100 μL of TMPD solution, which was freshly prepared before each experiment. Alternatively, 1% ascorbic acid was added to the TMPD solution to reduce autooxidation^30^. The colorimetric reaction resulting from the oxidation of TMPD was then quantified using a spectrophotometer (OD_570_). The oxidase test was conducted also in the presence of sodium cyanide (100 μM) and sodium azide (250 μM)^14^,^19^ (Sigma-Aldrich). However, to account for the dual effect of formate with azide or cyanide, the culture incubation time was optimized to 15 min after assessing different time points. Negative controls included MH broth only, formate + TMPD, azide + TMPD, and cyanide + TMPD. Different concentrations of fumarate and succinate were included as additional controls to contrast with the impact of formate on oxidase activity. The samples were tested in triplicate and the experiment was repeated three times.

### Aerotolerance

The tolerance of *C. jejuni* 81–176 to aerobic (ambient oxygen) conditions in the presence of formate was assessed as described previously^27^,^31^,^32^. Briefly, *C. jejuni* growing under microaerobic conditions on MH agar plates were harvested and suspended in MH broth to achieve an OD_600_ of 0.1. Cultures (100 mL) were transferred to different 250 mL sterile Erlenmeyer flasks covered with cotton plugs and were incubated under microaerobic conditions (200 rpm and 42 °C for an additional 2 h). Different concentrations of formate were added to each flask (t = 0 h post formate treatment) and the cultures were then incubated shaking (300 rpm) under ambient oxygen conditions at 42 °C. Aerotolerance was assessed by measuring OD_600_ using 1 mL plastic cuvettes and a spectrophotometer and counting CFU (as described above) at 8 h post formate treatment. Samples were tested in triplicate and the experiment was performed three times.

### qRT-PCR analysis

The expression of genes encoding respiratory proteins (electron donors and acceptors) and ribonucleotide reductase (genes and primers are listed in Table 1) was investigated in the presence and absence of formate (5 mM) and nitrate (1 mM) under microaerobic and anaerobic conditions, respectively. For this purpose, *C. jejuni* cultures were incubated for 15 min with shaking (200 rpm) at 42 °C^32^,^33^. The cells were then pelleted by centrifugation and RNA was extracted using RNeasy Mini Kit (Qiagen, CA, USA). cDNA was synthesized using SuperScript III First-Strand Synthesis SuperMix (Invitrogen, USA). The qPCR analysis was performed using SensiMixPlus SYBR RT-PCR Kit (Quantace, CA, USA). Gene expression was normalized with that of the 16S rRNA gene (internal control) and was compared to growth in MH broth only (no added formate and/or nitrate). Relative gene expression was determined using the 2^(−ΔΔC(T))^ method^34^. Gene expression studies were performed twice and samples were tested in triplicate in each experiment.

### Statistical analysis

Data were expressed as mean ± standard deviation and statistical analysis was performed using the one-way analysis of variance (ANOVA) followed by Tukey’s posttest. A *P* value of <0.05 was considered statistically significant.

## Results

### Formate is an important respiratory substrate and chemoattractant for *C. jejuni*

**Figure 1:**
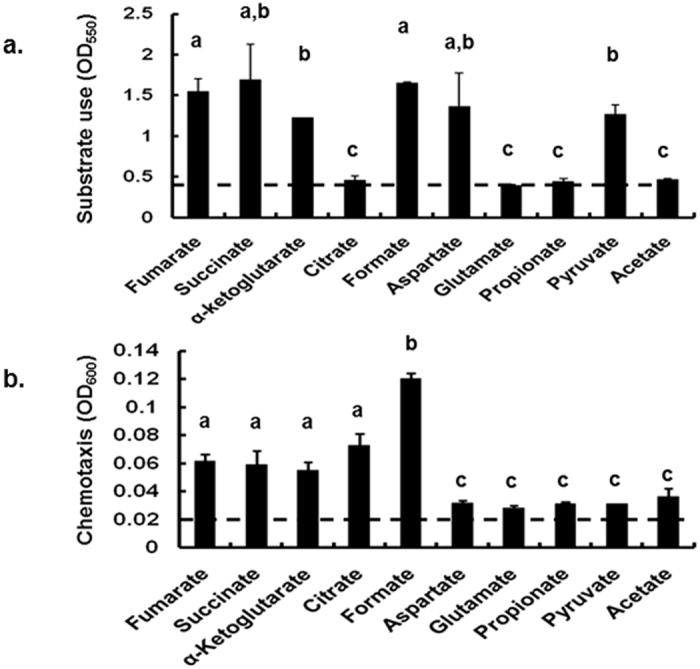
(**a)** Respiration of *C. jejuni* 81–176 in response to different substrates (organic acids). Formate is one of the substrates that are highly metabolized by *C. jejuni* in the BIOLOG-AN system. (**b)** Chemotaxis of *C. jejuni* 81–176 in response to different substrates was determined as described in Zhulin *et al*.^26^. Different letters indicate statistically significant differences (*P* < 0.05). Horizontal dashed line indicates the lowest limit for quantification in these assays.

In order to compare the metabolism of formate to that of other important substrates (salts of organic acids), *C. jejuni*’s respiration/ metabolism of these substrates was assessed using the BIOLOG-AN plates. Formate, succinate, fumarate, and aspartate were among the highly metabolized substrates (Fig. 1a). In these plates, pyruvate and α-ketoglutarate were also metabolized but to a significantly lesser extent (*P* < 0.05) in comparison to formate and fumarate (Fig. 1a). The metabolism of citrate, glutamate, propionate, and acetate was close to the assay’s detection limit under the experimental conditions used in this study (Fig. 1a).

To further establish the importance of formate as a respiratory substrate, the chemotaxis response of *C. jejuni* towards the aforementioned chemicals was evaluated. Under our experimental conditions, *C. jejuni* exhibited a significantly higher (*P* < 0.05) chemoattraction towards formate in comparison to all the other substrates (Fig. 1b). Although chemoattraction towards fumarate, succinate, α-ketoglutarate, and citrate was lower than the response to formate, it was significantly higher (*P* < 0.05) in comparison to the other tested substrates (Fig. 1b). Using the method described by Zhulin *et al*.^26^, we found that chemoattraction towards aspartate, glutamate, propionate, acetate, and pyruvate was the lowest and close to the assay’s detection limit (Fig. 1b).

### Formate enhances *C. jejuni*’s growth and motility

Since formate is a primary energy source for *C. jejuni* and bacterial growth and motility require energy production, the impact of formate on these phenotypes was investigated.

**Figure 2:**
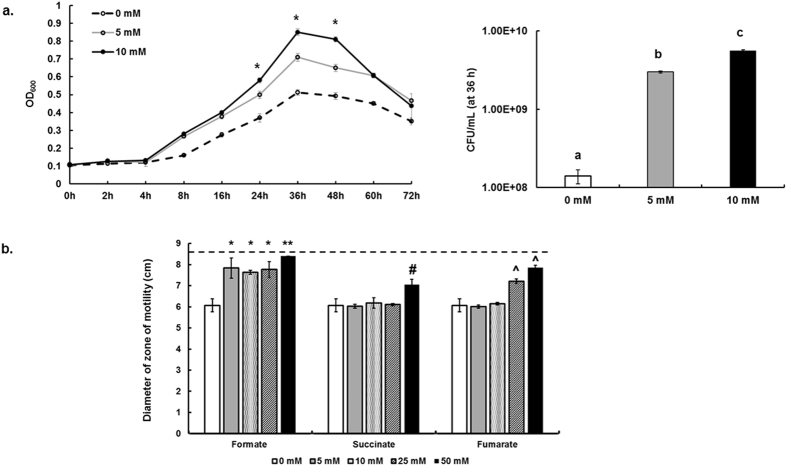
(**a)** Growth of *C. jejuni* 81–176 in Mueller-Hinton broth supplemented with different concentrations of formate. Growth was determined by measuring OD_600_. CFU numbers were quantified at 36 h. Asterisks and different letters indicate statistically significant differences between all treatments (*P* < 0.05). (**b)** Motility of *C. jejuni* 81–176 in Mueller-Hinton semi-solid (0.4%) agar supplemented with different concentrations of formate, succinate, and fumarate, respectively. The diameter of zone of motility was measured after 48 h under microaerobic conditions. Note that the diameter of the Petri dish was approximately 8.5 cm, which precluded assessment of the diameter beyond 8.5 cm. Horizontal dashed line indicates the maximum limit of quantification in this assay. Symbols indicate statistically significant differences (P < 0.05) in comparison to the control (0 mM). **Denotes the most significant increase (P < 0.05) in motility across all treatments.

Supplementation of MH broth with formate significantly increased (*P* < 0.05) the growth (OD_600_) of *C. jejuni* (Fig. 2a). However, the highest increase in growth was recorded after the addition of 10 mM formate, which increased the number of CFU/mL by ~40 times as compared to growth in MH broth only after 36 h of incubation (Fig. 2a). However, significant increase in OD_600_ in response to formate was not noted in the minimum essential medium, MEMα (data not shown).

Supplementation of semi-solid MH agar with formate significantly increased (*P* < 0.05) *C. jejuni*’s zone of motility regardless of the added formate concentration (Fig. 2b). In comparison, the zone of motility significantly increased (*P* < 0.05) only with relatively higher concentrations of succinate (50 mM) and fumarate (25 and 50 mM), respectively (Fig. 2b). The most significant increase in the zone of motility across all treatments was observed in the presence of 50 mM formate (Fig. 2b).

### Formate differentially impacts *C. jejuni*’s biofilm formation under different oxygen conditions

**Figure 3:**
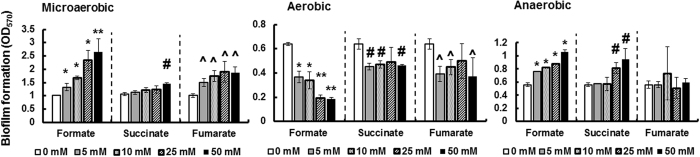
Biofilm formation of *C. jejuni* 81–176 in Mueller-Hinton broth supplemented with different concentrations of formate, succinate, and fumarate, respectively. Biofilm formation assays were performed using borosilicate tubes and crystal violet staining. After staining, the biofilms were suspended in DMSO (80%) and quantified using a spectrophotometer. Microaerobic: 5% O_2_; Anaerobic: <1% O_2_; Aerobic: ambient O_2_. Symbols indicate statistically significant differences (P < 0.05) in comparison to the control (0 mM). **Denotes the highest increase or decrease in biofilm formation across all treatments.

Biofilm formation in *C. jejuni* was associated with increased expression of the flagellar motility complex^35^. Therefore, biofilm formation was investigated in the presence of different concentrations of formate. Under microaerobic conditions, there was a significant dose-dependent increase (*P* < 0.05) in biofilm formation in response to the addition of formate (Fig. 3). However, there was no significant difference (*P* > 0.05) in biofilm formation between *C. jejuni* cultures supplemented with 25 and 50 mM formate. In contrast, a significant increase in biofilm formation (*P* < 0.05) was only observed with the highest concentration of succinate (50 mM). The addition of 5 mM fumarate resulted in a significant increase (*P* < 0.05) in biofilm formation. However, there was no significant difference (*P* > 0.05) in biofilm formation between *C. jejuni* cultures supplemented with 5, 10, 25, and 50 mM fumarate.

Under aerobic conditions, there was a significant dose-dependent decrease (*P* < 0.05) in biofilm formation in response to the addition of formate (Fig. 3). However, there was no significant difference (*P* > 0.05) in biofilm formation between *C. jejuni* cultures supplemented with either 5 and 10 mM formate or 25 and 50 mM formate. A significant decrease in biofilm formation (P < 0.05) was observed in response to 5 mM succinate and 5 mM fumarate, respectively. However, higher concentrations of succinate and fumarate did not significantly (*P* > 0.05) result in a further decrease in biofilm formation (Fig. 3).

Under anaerobic conditions, there was a significant dose-dependent increase (*P* < 0.05) in biofilm formation in response to the addition of formate (Fig. 3). However, there was no significant difference (*P* > 0.05) in biofilm formation between *C. jejuni* cultures supplemented with 5 and 10 mM formate. A significant increase in biofilm formation (*P* < 0.05) was observed in response to 25 and 50 mM succinate. Biofilm formation was not significantly (*P* > 0.05) affected by any of the tested fumarate concentrations (Fig. 3).

### Formate enhances the respiration of *C. jejuni*

**Figure 4:**
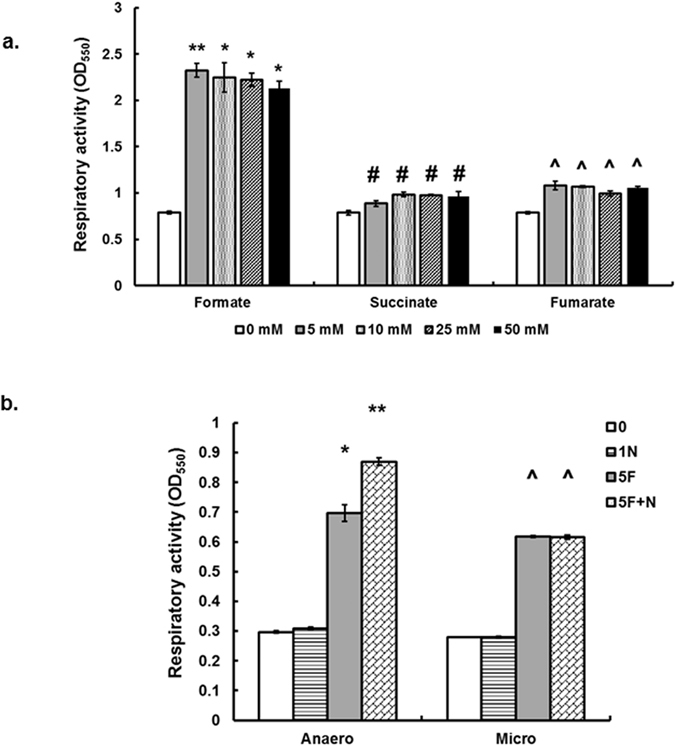
(**a)** Respiratory activity of *C. jejuni* 81–176 in Mueller-Hinton broth supplemented with different concentrations of formate, succinate, or fumarate under microaerobic conditions.. The respiratory activity was determined using CTC (5-cyano-2,3-ditolyltetrazolium chloride), which is reduced to formazan. This colorimetric reaction was then quantified using a spectrophotometer. (**b)** Respiratory activity of *C. jejuni* 81–176 in Mueller-Hinton broth supplemented with formate and nitrate. **Anaero:** <1% O_2_; **Micro:** 5% O_2_. **0:** No formate added; **1N:** Only 1 mM nitrate added; **5 F:** 5 mM formate added; **5F + N:** 5 mM formate and 1 mM nitrate added. Symbols indicate statistically significant differences (P < 0.05) in comparison to the control (No formate added). **Denotes the highest increase in respiration across all treatments.

Formate concentrations have been previously estimated in the mammalian hosts’ gut^5^,^36^,^37^. For example, formate concentrations ranged between an average of 3.2 and 8.2 mM in the intestines of mice^5^. Therefore, it was important to assess the respiration activity of *C. jejuni* in the presence of different concentrations of formate. All the tested formate concentrations significantly increased (*P* < 0.05) respiration by ~4 times in comparison to cultures in MH broth only (0 mM formate added). The highest activity was observed with 5 mM formate (Fig. 4a), while a slight but significant decrease (*P* < 0.05) in activity was observed with 50 mM formate in comparison to 5 mM formate. Regardless of the concentration, the respiratory activity in the presence of formate was significantly higher in comparison to succinate and fumarate (Fig. 4a). The addition of nitrate (1 mM) in the presence of formate (5 mM) significantly increased (*P* < 0.05) the respiratory activity under anaerobic conditions but not under microaerobic conditions (Fig. 4b). The latter was also observed when MH broth was replaced with MEMα (data not shown).

### Formate decreases oxidase activity and the ability of *C. jejuni* to tolerate ambient oxygen

**Figure 5:**
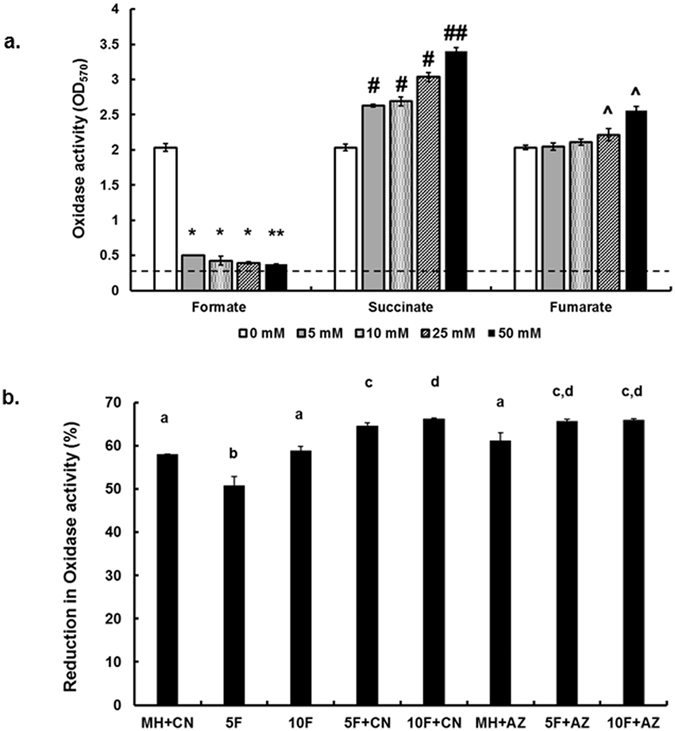
(**a)** Oxidase activity of *C. jejuni* 81–176 in Mueller-Hinton broth supplemented with different concentrations of formate, succinate, and fumarate, respectively. Oxidase activity was determined using TMPD (N,N,N′,N′-tetramethyl-p-phenylenediamine), a redox indicator, which is oxidized to form a dark blue color. The colorimetric reaction was then quantified using a spectrophotometer. Symbols indicate statistically significant differences (P < 0.05) in comparison to the control (0 mM). **Denoted the lowest decrease in oxidase activity across all treatments. ^##^Denoted the highest increase in oxidase activity across all treatments. Horizontal dashed line indicates the lowest limit for quantification in this assay. (**b)** Oxidase activity in the presence of formate, sodium azide and cyanide (inhibitors of oxidase activity). CN: cyanide; AZ: azide; 5F: 5 mM formate; 10F: 10 mM formate. Different letters indicate statistically significant differences (P < 0.05). ^d^Denotes the highest reduction in oxidase activity.

It was reported that formate inhibits oxidase activity in some bacterial species^20^,^21^. Therefore, the impact of formate on oxidase activity was investigated in *C. jejuni*. The addition of formate significantly (*P* < 0.05) decreased oxidase activity; however, the highest reduction was observed with 50 mM formate (Fig. 5a). In comparison, the addition of succinate significantly increased oxidase activity in a dose dependent manner (Fig. 5a). A significant increase (*P* < 0.05) in oxidase activity occurred with 25 and 50 mM fumarate but not with other fumarate concentrations. Similar results were obtained when MH broth was replaced with MEMα (data not shown).

The addition of cyanide and azide reduced oxidase activity to a level that was comparable to 10 mM formate (Fig. 5b). The addition of cyanide and azide in the presence of formate significantly reduced (*P* < 0.05) oxidase activity in comparison to cultures with formate, cyanide, or azide only (Fig. 5b).

**Figure 6:**
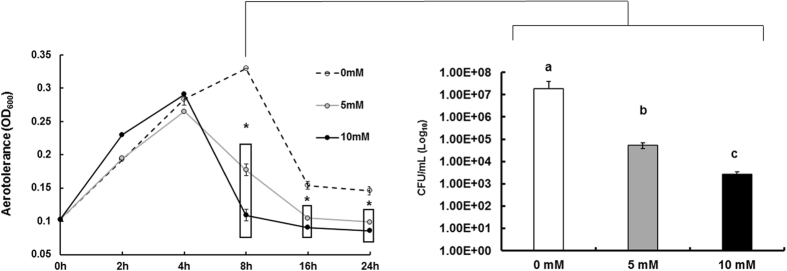
Aerotolerance of *C. jejuni* 81–176 in Mueller-Hinton broth supplemented with different concentrations of formate. The cultures were shifted from microaerobic conditions to ambient oxygen conditions with shaking (300 rpm). OD_600_ was measured at different time points and CFU numbers were quantified at 8 h of incubation under ambient oxygen. Asterisk * indicates statistically significant differences (*P* < 0.05) in comparison to the control (0 mM). Different letters indicate statistically significant differences (*P* < 0.05).

To further assess the impact of formate on phenotypes that might be associated with oxidase activity, aerotolerance in the presence of formate was measured. Aerotolerance in the presence of formate (5 and 10 mM) was significantly reduced after 8 h of exposure to ambient conditions (Fig. 6). Neither succinate nor fumarate (10 mM) significantly decreased (P > 0.05) tolerance to ambient oxygen (data not shown).

### Formate increases the expression of genes encoding RNR and respiratory proteins that facilitate the use of alternative electron acceptors

**Figure 7:**
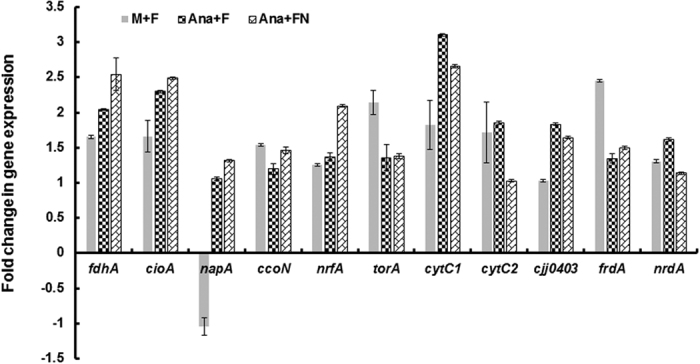
Fold change in gene expression in response to the addition of formate and nitrate under different oxygen concentrations. The expression was normalized using the 16S rRNA gene as well as by comparison to expression in MH broth only. Fold change was determined using the 2^(−ΔΔC(T))^ method^34^. **Ana:** < 1% O_2_; **Micro:** 5% O_2_. **F:** 5 mM formate; **FN:** 5 mM formate + 1 mM nitrate.

To further understand the impact of formate on respiration and survival under different oxygen conditions, we investigated the expression of selected genes (Table 1) encoding RNR, formate dehydrogenase, terminal oxidase and respiratory proteins that are associated with alternative electron acceptors in response to formate. Under microaerobic conditions, the addition of formate increased the expression of all tested genes with the exception of *napA* (nitrate reductase), which was downregulated (Fig. 7). Under anaerobic conditions, the expression of *fdhA, cioA, napA, cytC1, cjj81176_0403*, and *nrdA* significantly increased (*P* < 0.05) in response to formate in comparison to microaerobic conditions. Furthermore, the addition of formate and nitrate under anaerobic conditions significantly increased the expression of *fdhA, cioA, napA, nrfA*, and *cjj81176_0403* in comparison to growth in a medium supplemented with formate only (Fig. 7).

## Discussion

*C. jejuni* colonizes the gut, a complex niche that is influenced by the activity of the host’s microbiota which contribute to the development of an oxygen-limited environment and various metabolic byproducts^38^,^39^. The highly-branched respiratory chain of *C. jejuni* has been highlighted as a notable feature that facilitates the use of oxygen and alternative compounds as terminal electron acceptors^18^. In addition, *C. jejuni* can metabolize a variety of substrates, including fermentation byproducts, as electron donors to generate energy^18^. Subsequently, the respiratory chain enables *C. jejuni* to generate energy and persist in niches where oxygen is relatively scarce such as the hosts’ gut. However, how *C. jejuni* optimizes and coordinates electron transport to facilitate respiration in response to a niche’s properties (e.g. available substrates and oxygen concentrations) is not entirely clear. Here, we hypothesized that physiological concentrations of formate simultaneously enhance respiration and reduce oxidase activity. This is a previously undescribed mechanism in *C. jejuni* that might shed a light on the adaptation of this pathogen to oxygen-limitation. We suggest that the formate-dependent reduction in oxidase activity (Fig. 5) might serve in: (1) allowing the shuttling of electrons to alternative electron acceptors (Fig. 7), and (2) save the scarce oxygen in the gut for other functions such as DNA synthesis via the ribonucleotide reductase (RNR) (Fig. 7). The latter is notable, because *C. jejuni* contains a single class I-type RNR that requires oxygen to generate a tyrosyl radical, an observation that was associated with impeded DNA synthesis and cognate lack of *C. jejuni* growth under strictly anaerobic conditions^23^. In comparison, *E. coli* possesses an oxygen-sensitive class III-type ribonucleoside-triphosphate reductase that facilitates the production of deoxyribonucleoside triphosphates during anaerobic growth^40^,^41^. Remarkably, formate was identified as a hydrogen donor for this anaerobic ribonucleotide reductase in *E. coli*^41^.

Formate is an important metabolic substrate for *C. jejuni*. We showed that formate is a strong chemoattractant and a preferred respiratory substrate (Figs 1 and 4a), corroborating the observations of others^14^,^15^,^16^. However, the concentrations of substrates in the BIOLOG plates are generally high (>100 mM)^42^, and the physiological concentrations of formate in the mammalian host are estimated to be significantly lower^5^,^36^,^37^. Therefore, it was crucial to use physiologically relevant concentrations of formate (5–10 mM)^5^ to better evaluate the impact on *C. jejuni*’s respiration and other phenotypes in comparison to other important metabolites such as fumarate and succinate^12^,^18^. Notably the highest respiratory activity in *C. jejuni* was recorded in the presence of 5 mM formate (Fig. 4a). In comparison, respiration in the presence of succinate or fumarate (even at 50 mM) was significantly lower (Fig. 4a). This further supported the role of formate as a major energy source for *C. jejuni*.

Physiologically relevant concentrations of formate significantly enhanced important survival phenotypes in *C. jejuni*, namely growth, motility, and biofilm formation under microaerobic conditions (Figs 2 and 3). Increase in *C. jejuni* growth and motility was the highest at 10 mM and 50 mM formate, respectively (Fig. 2). This was not surprising, because it was shown that the addition of formate to MH broth significantly reduced the generation time of *C. jejuni* NCTC-11168^13^. Specifically, the generation times after the addition of 10 and 25 mM formate were 0.76 ± 0.07 h and 0.94 ± 0.11 h in comparison to 2.40 ± 0.17 h in MH broth only^13^. We also noted that the addition of formate did not result in significant growth in minimal media (data not shown), which suggested that formate was not used as a source of carbon but only energy. The positive impacts on growth (in supplemented MH broth) and respiration support the increase in motility and biofilm formation observed in our study (Figs 2 and 3). This is not surprising, because (1) bacterial biofilm formation and motility are energy requiring processes^43^,^44^, and (2) motility affects biofilm formation in *C. jejuni*^35^,^45^. The aforementioned positive impacts of formate on *C. jejuni* suggest that this metabolite might play an important role in the pathobiology of *C. jejuni*. While the impact of formate remains to be assessed *in vivo*, previous studies showed that the impairment of formate dehydrogenase reduced motility, biofilm formation, and the ability to infect host intestinal cells *in vitro*^10^,^11^ and colonize chickens^12^,^13^.

Physiological concentrations of formate significantly reduced oxidase activity of *C. jejuni* (Fig. 5a). While this was expected based on previous studies in other bacteria^20^,^21^, the extent of the reduction in oxidase activity in the presence of formate, even at the lower concentrations, was very notable. To further investigate the potential ramifications associated with reduction in oxidase activity, we tested aerotolerance and biofilm formation in the presence of formate under ambient oxygen conditions. We observed a significant decrease in both phenotypes (Figs 3 and 6) under these conditions. In fact, there was a gradual decrease in biofilm formation in response to increasing formate concentrations (Fig. 3) under ambient oxygen conditions. This was strikingly opposite to the biofilm formation in response to formate under microaerobic and anaerobic conditions, respectively (Fig. 3). This indicated an increased susceptibility of *C. jejuni* to ambient oxygen levels in the presence of formate, which was probably associated with the reduced oxidase activity. However, in our study, formate enhanced *C. jejuni*’s respiration and growth-associated phenotypes under microaerobic (5% O_2_) conditions. The latter is consistent with observations that a *cioAB* mutant did not have a significant defect in growth under 5% and 10% O_2_ in comparison to wildtype^19^, while a *ccoN* mutant was designated as O_2_-sensitive and could form colonies only under <7% oxygen conditions^22^. In fact, it was reported that formate enhanced growth of *C. sputorum* subsp. *bubulus*, a microaerophilic bacterium, at low dissolved oxygen tensions but the growth slowed down at higher oxygen tensions^30^. Therefore, a significant decrease in oxidase activity might be detrimental to *C. jejuni* at higher oxygen conditions (>5% O_2_)^22^. The increase in biofilm formation under anaerobic conditions in response to formate (Fig. 3) further supports the previous observations. Interestingly, the addition of formate (5 and 10 mM) in combination with either cyanide or azide, inhibitors of cytochrome oxidases^14^,^19^, resulted in further reduction in oxidase activity as compared to formate alone (Fig. 5b), suggesting that this metabolite might be acting on both terminal oxidases. The latter might be true considering that CioAB is cyanide-insensitive, while CcoNOQP is cyanide-sensitive^19^.

Our data suggested that the presence of formate can enhance the use alternative terminal electron acceptors, which is especially important in a niche that is oxygen limited and rich in anaerobic metabolites such as the gut. This was especially evident as the highest respiration was noted in the presence of formate and nitrate under anaerobic conditions which aligns with the expression data (Figs 4b and 7). The increase in the expression of *cioA* and *ccoN* in response to formate, especially under anaerobic conditions, was not expected. However, this might further indicate that the inhibition of oxidase activity by formate occurs on the enzymatic level. In a previous study, it was shown that formate binds to and inhibits cytochrome c oxidase in submitochondrial particles^46^. Furthermore, the increase in the oxidase gene expression might be a compensatory response to the formate-dependent inhibition of the oxidase activity. It was notable that the expression of *nrdA* increased in compensatory response to formate, which is crucial for DNA synthesis and the survival of *C. jejuni*^23^. Taken together, we propose that in an oxygen-limited niche like the gut, formate might act to reduce the oxidase activity, shuttling electrons to alternative electrons acceptors, while sparing the scarce oxygen for the essential RNR activity, whose expression is also enhanced in the presence of formate (Fig. 7). This might constitute a significant and previously undescribed adaptation that facilitates the notable success of *C. jejuni* in colonizing the gut.

The gut is a complex niche(s), occupied by a diverse microbiota and metabolites and challenging conditions. *C. jejuni*, a bacterium with a relatively small genome, must possess the necessary adaptations and survival strategies to cope with and exploit this environment. Therefore, using a metabolite such as formate both as an energy generating substrate and a possible signal to optimize energy and survival constitutes an advantage and sheds a new insight on how *C. jejuni* interacts with its environment. It should be also noted that other gut metabolites might modulate the impact of formate, which further highlights the complexity of the interaction between a pathogen and its host’s gut.

## Additional Information

**How to cite this article**: Kassem, I. I. *et al*. Formate simultaneously reduces oxidase activity and enhances respiration in *Campylobacter jejuni. Sci. Rep.* **7**, 40117; doi: 10.1038/srep40117 (2017).

**Publisher's note:** Springer Nature remains neutral with regard to jurisdictional claims in published maps and institutional affiliations.

## Figures and Tables

**Table 1:** List of genes that were targeted for qRT-PCR analysis.

Target Gene (encoded respiratory protein/subunit)	Primer Sequence
*fdhA* (formate dehydrogenase)*	5′-GGCTTAGTTCCTTCTGGGAAAT-3′
	5′-GAGTGAAGGTTAATCCAGGTGTAG-3′
*frdA* (fumarate reductase)*^	5′-GCGGTAATTTGTGAAGGAACAG-3′
	5′-GGCACAATAGGAGTTGGATGA-3′
*napA* (nitrate reductase)^	5′-GATGAGCCATCAGGCTGTTAT-3′
	5′-CCAAAGGATTGGGTGCATTTC-3′
*nrfA* (nitrite reductase)^	5′-ATCCGCTCTCATTTGAGCTTTA-3′
	5′-CGCGATGAGCTAGGTAATATGG-3′
*torA* (trimethylamine N-oxide reductase)^	5′-GCCAGTGGTGTAAGTGTCTAA-3′
	5′-ATAGCTCCACGCCCAAATAC-3′
*cioA* (cyanide-insensitive oxidase)^	5′-GGTGTGTGGCTATAGGAAGTAATC-3′
	5′-TCTTGCTGTGTCAGGGTTAAAG-3′
*ccoN (cb*-type oxidase)^	5′-AGCGCAGCCATAGTCATAAA-3′
	5′-CACTTGAAGGGCCAATTCTTTC-3′
*cytC-1 (cyt c* peroxidase)^	5′-CGCCCTAAATCTTCTTGGTTTG-3′
	5′-TGGCTGCATCTCTTGTCATC-3′
*cytC-2 (cyt c* peroxidase)^	5′-TGGACAGCAAATCCTCATCAT-3′
	5′-TCAGCTTGGATTGGACCTTTAG-3′
*CJJ81176_0403* (putative oxidoreductase)^	5′-CCTACTGCAAGCAAGGTAAGA-3′
	5′-CCCGATCAAGACGCTTTATTTC-3′
*nrdA* (ribonucleotide reductase)	5′-CCTCAAGAATGGGCGAAGAA-3′
	5′-GCGCGTTGTTCTTGCATTAG-3′

The primers were designed using the PrimerQuest Tool (Integrated DNA Technologies). ^*^Respiratory proteins that facilitate the transfer of electrons from electron donors. ^^^Respiratory proteins that facilitate the transfer of electrons to electron acceptors. This list includes the majority of previously described respiratory acceptor proteins in *C. jejuni*^12^,^18^.

## References

[b1] Altekruse S. F., Stern N. J., Fields P. I. & Swerdlow D. L. *Campylobacter jejuni*–an emerging foodborne pathogen. Emerg. Infect. Dis. 5, 28–35 (1999). 10081669 10.3201/eid0501.990104 PMC2627687

[b2] Sahin O. . *Campylobacter* in poultry: ecology and potential interventions. Avian Dis. 59, 185–200 (2015). 26473668 10.1637/11072-032315-Review

[b3] Hoffmann S., Batz M. B. & Morris J. G. Jr. Annual cost of illness and quality-adjusted life year losses in the United States due to 14 foodborne pathogens. J. Food Prot. 75, 1292–1302 (2012). 22980013 10.4315/0362-028X.JFP-11-417

[b4] Vogt S. L., Peña-Díaz J. & Finlay B. B. Chemical communication in the gut: effects of microbiota-generated metabolites on gastrointestinal bacterial pathogens. Anaerobe 34, 106–115 (2015). 25958185 10.1016/j.anaerobe.2015.05.002

[b5] Huang Y., Suyemoto M., Garner C. D., Cicconi K. M. & Altier C. Formate acts as a diffusible signal to induce *Salmonella* invasion. J. Bacteriol. 190, 4233–4241 (2008). 18424519 10.1128/JB.00205-08 PMC2446767

[b6] Macfarlane G. T., Gibson G. R. & Cummings J. H. Comparison of fermentation reactions in different regions of the human colon. J. Appl. Bacteriol. 72, 57–64 (1992). 1541601 10.1111/j.1365-2672.1992.tb04882.x

[b7] Lawhon S. D., Maurer R., Suyemoto M. & Altier C. Intestinal short‐chain fatty acids alter *Salmonella typhimurium* invasion gene expression and virulence through BarA/SirA. Mol. Microbiol. 46, 1451–1464 (2002). 12453229 10.1046/j.1365-2958.2002.03268.x

[b8] Kirkpatrick C. . Acetate and formate stress: opposite responses in the proteome of Escherichia coli. J. Bacteriol. 183, 6466–6477 (2001). 11591692 10.1128/JB.183.21.6466-6477.2001 PMC100143

[b9] Barker H. C., Kinsella N., Jaspe A., Friedrich T. & O’Connor C. D. Formate protects stationary‐phase *Escherichia coli* and *Salmonella* cells from killing by a cationic antimicrobial peptide. Mol. Microbiol. 35, 1518–1529 (2000). 10760151 10.1046/j.1365-2958.2000.01820.x

[b10] Pryjma M., Apel D., Huynh S., Parker C. T. & Gaynor E. C. FdhTU-modulated formate dehydrogenase expression and electron donor availability enhance recovery of *Campylobacter jejuni* following host cell infection. J. Bacteriol. 194, 3803–3813 (2012). 22636777 10.1128/JB.06665-11 PMC3416569

[b11] Kassem I. I. . Respiratory proteins contribute differentially to *Campylobacter jejuni*’s survival and *in vitro* interaction with hosts’ intestinal cells. BMC Microbiology 12, 258 (2012). 23148765 10.1186/1471-2180-12-258 PMC3541246

[b12] Hitchcock A. . Roles of the twin-arginine translocase and associated chaperones in the biogenesis of the electron transport chains of the human pathogen *Campylobacter jejuni*. Microbiology 156, 2994–3010 (2010). 20688826 10.1099/mic.0.042788-0

[b13] Weerakoon D. R., Borden N. J., Goodson C. M., Grimes J. & Olson J. W. The role of respiratory donor enzymes in *Campylobacter jejuni* host colonization and physiology. Microb. Pathog. 47, 8–15 (2009). 19397993 10.1016/j.micpath.2009.04.009

[b14] Vegge C. S., Brøndsted L., Li Y. P., Bang D. D. & Ingmer H. Energy taxis drives *Campylobacter jejuni* toward the most favorable conditions for growth. Appl. Environ. Microbiol. 75, 5308–5314 (2009). 19542337 10.1128/AEM.00287-09 PMC2725471

[b15] Tareen A. M., Dasti J. I., Zautner A. E., Groß U. & Lugert R. *Campylobacter jejuni* proteins Cj0952c and Cj0951c affect chemotactic behaviour towards formic acid and are important for invasion of host cells. Microbiology 156, 3123–3135 (2010). 20656782 10.1099/mic.0.039438-0

[b16] Hoffman P. S. & Goodman T. G. Respiratory physiology and energy conservation efficiency of *Campylobacter jejuni*. J. Bacteriol. 150, 319–326 (1982). 6277867 10.1128/jb.150.1.319-326.1982 PMC220116

[b17] Kassem I. I. & Rajashekara G. Formate Dehydrogenase Localization and Activity Are Dependent on an Intact Twin Arginine Translocation System (Tat) In *Campylobacter jejuni* 81–176. Foodborne Pathog. Dis. 11, 917–919 (2014). 25268895 10.1089/fpd.2014.1797

[b18] Kelly D. Complexity and Versatility in the Physiology and Metabolism of Campylobacter jejuni in Campylobacter, Third Edition (ed. Nachamkin I., Szymanski C. & Blaser M.) 41–61 (ASM Press, 2008).

[b19] Jackson R. J. . Oxygen reactivity of both respiratory oxidases in *Campylobacter jejuni*: the cydAB genes encode a cyanide-resistant, low-affinity oxidase that is not of the cytochrome bd type. J. Bacteriol. 189, 1604–1615 (2007). 17172349 10.1128/JB.00897-06 PMC1855770

[b20] Ivanovsky R. N., Zacharova E. V., Netrusov A. I., Rodionov Y. V. & Kondratieva E. N. The effect of formate on oxidase activities in different bacteria. FEMS Microbiol. Lett. 8, 139–142 (1980).

[b21] Sharpe M., Perin I., Tattrie B. & Nicholls P. Ligation, inhibition, and activation of cytochrome c oxidase by fatty acids. Biochem. Cell Biol. 75, 71–79 (1997). 9192076

[b22] Weingarten R. A., Grimes J. L. & Olson J. W. Role of *Campylobacter jejuni* respiratory oxidases and reductases in host colonization. Appl. Environ. Microbiol. 74, 1367–1375 (2008). 18192421 10.1128/AEM.02261-07 PMC2258625

[b23] Sellars M. J., Hall S. J. & Kelly D. J. Growth of *Campylobacter jejuni* supported by respiration of fumarate, nitrate, nitrite, trimethylamine-N-oxide, or dimethyl sulfoxide requires oxygen. J. Bacteriol. 184, 4187–4196 (2002). 12107136 10.1128/JB.184.15.4187-4196.2002 PMC135223

[b24] Hu L. & Kopecko D. J. *Campylobacter jejuni* 81–176 associates with microtubules and dynein during invasion of human intestinal cells. Infect. Immun. 67, 4171–4182 (1999). 10417189 10.1128/iai.67.8.4171-4182.1999 PMC96722

[b25] Brandl M. T., Haxo A. F., Bates A. H. & Mandrell R. E. Comparison of survival of *Campylobacter jejuni* in the phyllosphere with that in the rhizosphere of spinach and radish plants. Appl. Environ. Microbiol. 70, 1182–1189 (2004). 14766604 10.1128/AEM.70.2.1182-1189.2004 PMC348832

[b26] Zhulin I. B., Gibel I. B. & Ignatov V. V. A rapid method for the measurement of bacterial chemotaxis. Curr. Microbiol. 22, 307–309 (1991).

[b27] Fields J. A. & Thompson S. A. *Campylobacter jejuni* CsrA mediates oxidative stress responses, biofilm formation, and host cell invasion. J. Bacteriol. 190, 3411–3416 (2008). 18310331 10.1128/JB.01928-07 PMC2347403

[b28] Gangaiah D., Kassem I. I., Liu Z. & Rajashekara G. Importance of polyphosphate kinase 1 for *Campylobacter jejuni* viable-but-nonculturable cell formation, natural transformation, and antimicrobial resistance. Appl. Environ. Microbiol. 75, 7838–7849 (2009). 19837830 10.1128/AEM.01603-09 PMC2794102

[b29] Rezaeinejad S. & Ivanov V. Heterogeneity of *Escherichia coli* population by respiratory activity and membrane potential of cells during growth and long-term starvation. Microbiol. Res. 166, 129–135 (2011). 20171858 10.1016/j.micres.2010.01.007

[b30] Niekus H. G., Van Doorn E. L. S., De Vries W. Y. T. S. K. E. & Stouthamer A. H. Aerobic growth of *Campylobacter sputorum* subspecies *bubulus* with formate. Microbiology 118, 419–428 (1980). 10.1007/BF00428017 7425784

[b31] Atack J. M., Harvey P., Jones M. A. & Kelly D. J. The *Campylobacter jejuni* thiol peroxidases Tpx and Bcp both contribute to aerotolerance and peroxide-mediated stress resistance but have distinct substrate specificities. J. Bacteriol. 190, 5279–5290 (2008). 18515414 10.1128/JB.00100-08 PMC2493253

[b32] Kassem I. I. . The impairment of methylmenaquinol: fumarate reductase affects hydrogen peroxide susceptibility and accumulation in *Campylobacter jejuni*. MicrobiologyOpen 3, 168–181(2014). 24515965 10.1002/mbo3.158 PMC3996566

[b33] Palyada K. . Characterization of the oxidative stress stimulon and PerR regulon of *Campylobacter jejuni*. BMC Genomics 10, 481 (2009). 19835633 10.1186/1471-2164-10-481 PMC2772861

[b34] Livak K. J. & Schmittgen T. D. Analysis of relative gene expression data using real-time quantitative PCR and the 2^−ΔΔCT^ method. Methods 25, 402–408 (2001). 11846609 10.1006/meth.2001.1262

[b35] Kalmokoff M. . Proteomic analysis of *Campylobacter jejuni* 11168 biofilms reveals a role for the motility complex in biofilm formation. J. Bacteriol. 188, 4312–4320 (2006). 16740937 10.1128/JB.01975-05 PMC1482957

[b36] Stefanowicz A. The Biolog plates technique as a tool in ecological studies of microbial communities. Pol. J. Environ. Stud. 15, 669–676 (2006).

[b37] Payne A. N. . The metabolic activity of gut microbiota in obese children is increased compared with normal-weight children and exhibits more exhaustive substrate utilization. Nutr. Diabetes 1, e12 (2011). 23154580 10.1038/nutd.2011.8 PMC3302137

[b38] Albenberg L. . Correlation between intraluminal oxygen gradient and radial partitioning of intestinal microbiota. Gastroenterology 147, 1055–1063 (2014). 25046162 10.1053/j.gastro.2014.07.020 PMC4252572

[b39] Espey M. G. Role of oxygen gradients in shaping redox relationships between the human intestine and its microbiota. Free Radic. Biol. Med. 55, 130–140 (2013). 23127782 10.1016/j.freeradbiomed.2012.10.554

[b40] Mulliez E., Fontecave M., Gaillard J. & Reichard P. An iron-sulfur center and a free radical in the active anaerobic ribonucleotide reductase of *Escherichia coli*. J. Biol. Chem. 268, 2296–2299 (1993). 8381402

[b41] Mulliez E., Ollagnier S., Fontecave M., Eliasson R. & Reichard P. Formate is the hydrogen donor for the anaerobic ribonucleotide reductase from *Escherichia coli*. Proc. Natl. Acad. Sci. USA 92, 8759–8762 (1995). 7568012 10.1073/pnas.92.19.8759 PMC41046

[b42] Garland J. L., Roberts M. S., Levine L. H. & Mills A. L. Community-level physiological profiling performed with an oxygen-sensitive fluorophore in a microtiter plate. Appl. Environ. Microbiol. 69, 2994–2998 (2003). 12732576 10.1128/AEM.69.5.2994-2998.2003 PMC154488

[b43] Garrett T. R., Bhakoo M. & Zhang Z. Bacterial adhesion and biofilms on surfaces. Prog. Nat. Sci. 18, 1049–1056 (2008).

[b44] Lertsethtakarn P., Ottemann K. M. & Hendrixson D. R. Motility and chemotaxis in *Campylobacter* and *Helicobacter*. Annu. Rev. Microbiol. 65, 389–410 (2011). 21939377 10.1146/annurev-micro-090110-102908 PMC6238628

[b45] Pascoe B. . Enhanced biofilm formation and multi‐host transmission evolve from divergent genetic backgrounds in *Campylobacter jejuni*. Environ. Microbiol. 17, 4779–4789 (2015). 26373338 10.1111/1462-2920.13051 PMC4862030

[b46] Nicholls P. Formate as an inhibitor of cytochrome c oxidase. Biochem. Biophys. Res. Commun. 67, 610–616 (1975). 1020 10.1016/0006-291x(75)90856-6

